# Fibronectin-binding protein B variation in *Staphylococcus aureus*

**DOI:** 10.1186/1471-2180-10-160

**Published:** 2010-06-01

**Authors:** Fiona M Burke, Niamh McCormack, Simonetta Rindi, Pietro Speziale, Timothy J Foster

**Affiliations:** 1 Department of Microbiology, Moyne Institute of Preventive Medicine, University of Dublin, Trinity College, Dublin, Ireland; 2 Department of Biochemistry, University of Pavia, Pavia, Italy

## Abstract

**Background:**

Fibronectin binding proteins A and B (FnBPA and FnBPB) mediate adhesion of *S. aureus *to fibrinogen, elastin and fibronectin. We previously identified seven different isotypes of FnBPA based on divergence in the fibrinogen- and elastin-binding A domains. The variation created differences in antigenicity while ligand binding functions were retained. Here, FnBPB variation was examined in both human and bovine isolates and compared to that of FnBPA.

**Results:**

Seven different *fnbB *allelic variants were identified. Some strains that cluster by phylogenetic analysis contain different *fnbB *variants, whereas more divergent strains contain the same *fnb*B variant. The phylogeny of *fnb*B alleles does not match the phylogeny of *fnbA *alleles. Some FnBPA and FnBPB isotypes that are specified by human *S. aureus *strains are also found in bovine strains. The seven *fnb*B allelic variants encode seven distinct isotypes of the FnBPB A domain that are 61 to 85% identical in amino acid sequence. Variant amino acid residues were mapped on a three-dimensional model of the FnBPB A domain and were predicted to be surface-exposed. They are responsible for the antigenic diversity detected with polyclonal antibody and a monoclonal antibody raised against isotype I. Ligand binding by recombinant FnBPB N23 isotypes was compared by ELISA-based solid phase assays and surface plasmon resonance. Each bound to immobilized fibrinogen, elastin and fibronectin dose-dependently and saturably with similar affinities. Binding to fibronectin was surprising because the A domains do not contain any known motifs that mediate binding to fibronectin. This raises the possibility that the A domain of FnBPB contains a novel fibronectin binding motif that binds fibronectin by a novel mechanism.

**Conclusions:**

Seven different isoforms of FnBPB A domain retain ligand-binding functions but are antigenically distinct. The variation in FnBPA and FnBPB occurs in human and bovine *S. aureus *strains and may act as an immune evasion mechanism. All seven isotypes of FnBPB are capable of binding fibronectin though none contain any known fibronectin-binding motifs. These results have implications for the development of vaccines or immunotherapeutics that target FnBPB

## Background

*Staphylococcus aureus *is a commensal that colonizes the moist squamous epithelium of the human anterior nares. Twenty percent of the population are permanently colonised while the remainder are colonized intermittently [1]. It is an important opportunistic pathogen that can cause superficial skin infections as well as invasive life-threatening conditions such as septic arthritis and endocarditis [2]. The success of *S. aureus *as a pathogen can in part be attributed to the expression of cell surface protein receptors designated MSCRAMMs (microbial surface components recognizing adhesive matrix molecules) that interact specifically with proteins present in the host plasma and extracellular matrix [3]. MSCRAMMs act as virulence factors that allow *S. aureus *to adhere to the surface of host cells and to damaged tissue and help it to avoid phagocytosis by neutrophils [4-6]

**Figure 1:**
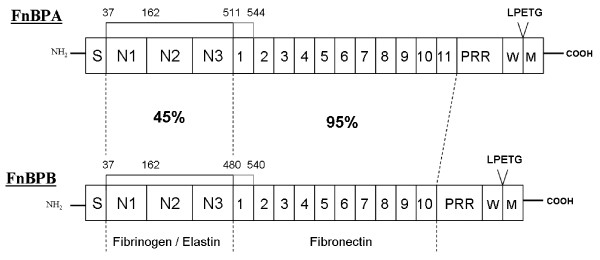
**Structural organisation of FnBPA and FnBPB from *S.aureus *8325-4**. The N-terminus of FnBPA and FnBPB contain a signal sequence (S) followed by a fibrinogen and elastin binding A domain consisting of subdomains N1, N2 and N3. Following the A domains are tandemly repeated fibronectin-binding motifs. The A domains as they were originally defined contain a single fibronectin-binding motif. The true A domains of FnBPA and FnBPB are now considered to include residues 37- 511 and residues 37-480, respectively. At the C-termini are proline-rich repeats (PRR), wall (W) and membrane (M)-spanning domains, and the sortase recognition motif LPETG. The percentage amino acid identities between the binding domains of FnBPA and FnBPB from *S.aureus *8325-4 are shown.

The fibronectin binding proteins (FnBPs) A and B of *S. aureus *are multifunctional MSCRAMMs which recognise fibronectin, fibrinogen and elastin [7-10]. FnBPA and FnBPB have considerable organization and sequence similarity and are composed of a number of distinct domains [7,9]. Figure 1 illustrates the domain organization of FnBPA and FnBPB of *S. aureus *strain 8325-4. Both proteins contain a secretory signal sequence at the N-terminus and a C-terminal LPETG motif required for sortase-mediated anchoring of the proteins to the cell wall peptidoglycan. The N-terminal A domains of FnBPA and FnBPB are exposed on the cell surface and promote binding to fibrinogen and elastin [10,11]. Based on their sequence similarity to the fibrinogen binding A domain of clumping factor A (ClfA) [12], the A domains of FnBPA and FnBPB are predicted to fold into three sub-domains N1, N2 and N3 similar to ClfA [13]. The A domains of FnBPA, FnBPB and ClfA bind fibrinogen at the C-terminus of the γ-chain [10,14]. Unlike ClfA, the A domains of FnBPA and FnBPB also bind to elastin [8]. It is proposed that ligand binding occurs through the same dynamic "dock, lock, latch" mechanism that has been predicted for fibrinogen binding to the A domain of ClfA [13]. The fibrinogen γ-chain peptide binds to a groove located between domains N2 and N3 in the apo form. C-terminal residues in domain N3 undergo a conformational change to bind adjacent to a β-strand in domain N2 forming an extra β-strand termed the latching peptide. This traps the fibrinogen peptide in the groove between N2 and N3 and locks it in place [15].

Located distal to the A domains of FnBPA and FnBPB are unfolded regions which contain multiple, tandemly arranged motifs (Figure 1) that bind to the N-terminal type I modules of fibronectin by a tandem beta-zipper mechanism [16]. The sequences of the fibronectin-binding motifs are highly conserved in FnBPA and FnBPB from strain 8325-4 (95% amino acid identity). By contrast the sequences of the fibrinogen and elastin binding A domains are more divergent (45% amino acid identity).

Most research on fibronectin-binding proteins has been preformed with FnBPA from strain 8325-4. It was reported previously that the A domain of FnBPA of *S. aureus *strain 8325-4 comprising residues 37-544 bound to immobilized elastin and to fibrinogen (Figure 1.) [8,10]. The A domain co-ordinates were revised recently and it was noted that the C- terminus of rFnBPA37-544 likely contained a single fibronectin-binding motif [16]. The ability of this protein to bind fibronectin was later confirmed [11]. In the same study, the revised A domain of FnBPA spanning residues 194-511 (Figure 1) was shown bind fibrinogen and elastin but not fibronectin. The minimum region of the FnBPA A domain needed for binding to fibrinogen and elastin is subdomains N23 (residues 194-511). The N1 sub-domain is not required for ligand binding [11].

The binding of FnBPs to fibronectin promotes the internalization of *S. aureus *into epithelial and endothelial cells which are not normally phagocytic [17,18]. FnBP-mediated invasion occurs through the formation of a fibronectin bridge between *S. aureus *and the α5β1 integrin [18]. This may promote bacterial dissemination from the bloodstream to internal organs and evasion of immune responses and antibiotics. This was convincingly demonstrated in a study of the role of FnBPA in experimental endocarditis where binding to both fibrinogen and fibronectin required. Binding of fibrinogen was required for initial colonization of thrombi on damaged valves and while binding to fibronectin was required for the infection to spread [19].

FnBPA and FnBPB are encoded by two closely linked but separately transcribed genes, *fnbA *and *fnbB *[7,9]. While most strains contain both genes, some strains contain only *fnbA *[20]. In strain 8325-4, studies with site-specific *fnbA *and *fnbB *insertion mutants showed that either FnBPA or FnBPB mediated adherence to immobilized fibronectin but there was no significant difference in adherence between wild type strains and single *fnb *mutants [21]. However, studies with clinical isolates suggested that strain associated with invasive diseases are significantly more likely to have two *fnb *genes [20].

Seven variants (isotypes I-VII) of FnBPA were identified based on divergence in the amino acid sequences of the minimal ligand-binding N23 sub-domains [22]. Each FnBPA isotype retained ligand-binding activity but were antigenically distinct. Modelling the 3D structures showed that the amino acid variation occurred in surface-exposed residues and not in those involved in ligand-binding [22].

The initial aim of this study was to characterize the A domain of FnBPB and to determine the extent of variation in the A domain. It was discovered that the A domain of all FnBPB isotypes had the ability to bind to fibronectin by a novel mechanism.

## Results

### *fnbB *gene variation in *S. aureus *whole-genome sequences

Previously we reported that the A domain of FnBPA from strain P1 varied substantially from that of strain 8325-4, sharing only 73.5% amino acid identity [11]. We then identified seven variants of FnBPA A domain (isotypes I-VII) based on divergence in the minimal ligand-binding N23 sub-domain. Each recombinant N23 variant was shown to retain ligand-binding function but was antigenically distinct [22]. This prompted us to investigate variation in the A domain of the second fibronectin-binding protein, FnBPB.

DNA encoding the entire FnBPB A domain of strain P1 was amplified by PCR and sequenced. The deduced amino acid sequence was compared with that of strain 8325-4 and the overall identity was 80%. The A domain sequences of FnBPB from published *S. aureus *genomes were compared to determine if diversity in this domain is common amongst *S. aureus *isolates. All of the sequenced strains, except strain MRSA252 and the bovine strain RF122, contain genes encoding both FnBPA and FnBPB. Strains MRSA252 and RF122 both encode the FnBPA protein. The amino acid sequence of the A domain of FnBPB from *S. aureus *strains 8325-4, COL, USA300, Mu50, MSSA476, N315, MW2 and P1 were compared by pair-wise alignments and the identities calculated. Strains that are closely related and belonging to the same clonal complex were found to share identical A domains. However, comparison of A domain sequences of strains from different sequence types revealed that significant diversity exists. While subdomain N1 is highly conserved in all strains (94-100% amino acid identity) the N2 and N3 domains from unrelated isolates are significantly more divergent. Based on the sequences of the N23 subdomains, four variants of FnBPB (isotypes I-IV) were identified that share 61.1 - 80.6% amino acid identity (Table 1).

**Table 1:** Percentage amino acid identities of A domain isotypes I - VII*.

	I	II	III	IV	V	VI	VII
**I**	100%	72.6%	61.1%	77.1%	68.8%	76.6%	74.4%
**II**	72.6%	100%	65.5%	80.6%	76.4%	73.5%	82.0%
**III**	61.1%	65.5%	100%	65.5%	60.7%	66.0%	66.2%
**IV**	77.1%	80.6%	62.2%	100%	78.3%	73.1%	73.7%
**V**	68.8%	76.4%	60.7%	78.3%	100%	71.2%	71.8%
**VI**	76.6%	73.5%	66.0%	73.1%	71.2%	100%	85.0%
**VII**	74.4%	82.0%	66.2%	73.7%	71.8%	85.0%	100%

* Pairwise alignments were performed using the amino acid sequences of the N23 sub-domains of the FnBPB A domain.

### DNA hybridization analysis using *fnbB *isotype-specific probes

**Figure 2:**
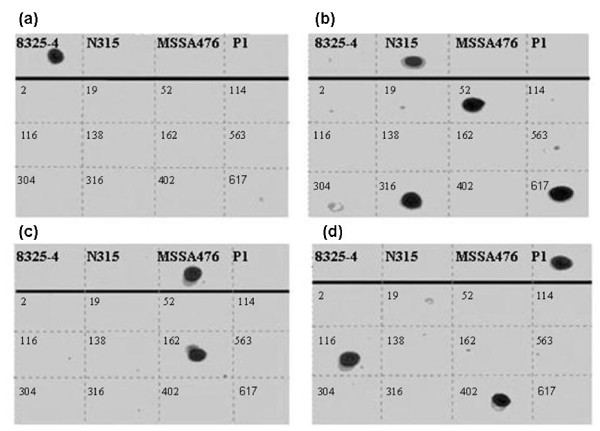
**FnBPB A domain typing of *S.aureus *strains by dot blot hybridisation**. DNA fragments coding for the entire A domain of *fnbB *were amplified by PCR from clinical *S.aureus *isolates. PCR products were spotted onto nitrocellulose membranes and probed with DIG-labelled probes specific for *fnbB *isotype I (A), II (B), III (C) and IV (D). *fnbB *DNA from strains 8325-4, N315, MSSA476 and P1 was used as control.

To determine the distribution of FnBPB A domain isotypes I - IV in *S. aureus *strains of different MLSTs and to identify any novel A domain isotypes, DNA hybridization was used with isotype-specific probes homologous to DNA specifying a portion of the highly divergent N3 sub-domain. DNA encoding the entire A domain was amplified with A domain flanking primers. PCR products were then spotted onto membranes and hybridized with the DIG-labelled type-specific probes. An example of the hybridization experiments with probes I - IV is shown in Figure 2. The probes were shown to be type-specific because each only hybridized to the appropriate control *fnbB *fragment (Figure 2A-D, top rows). *fnbB *DNA from *S. aureus *strains 2 (ST7),114 (ST39), 233 (ST45), 304 (ST39), 138 (ST30), 563 (ST37), 3077 (ST17) and 3110 (ST12) did not hybridise to any of the probes, indicating that they may specify novel FnBPB isotypes or lack the *fnbB *gene.

### Identification of novel FnBPB isotypes (Types V, VI and VII)

The *fnbB *gene fragments amplified from *S. aureus *strains 2 (ST7) 114 (ST39), 233 (ST45), 304 (ST39), 138 (ST30), 563 (ST37), 3077 (ST17) and 3110 (ST12) did not hybridise to probes specific for FnBPB isotypes I-IV. The *fnbB *gene fragments from these strains were cloned and sequenced, and the deduced A domain amino acid sequences were compared to the sequences of A domains of types I - IV. *S. aureus *strains 2 (ST7) and 3110 (ST12) specify a novel FnBPB A domain called isotype V (N23, 68.8 - 73.3% identical to isotypes I - IV). The A domains of strains 3077 (ST17) and 233 (ST45) are also different and are called isotype VI (N23, 66.0- 76.6% identical to types I - V) and isotype VII (N23, 66.2% - 85% identical to types I-VI) (Table 1). Strains 114, 563, 138 and 304 specify an identical A domain which is 92% identical to isotype II and is called isotype II* (Table 1)

### Phylogenetic analysis of FnBPB A domain isotypes I-VII

**Figure 3:**
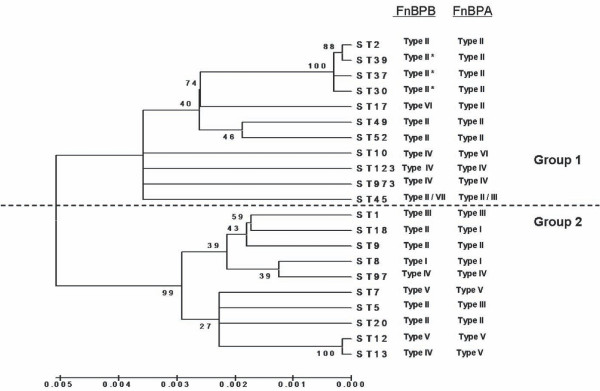
**Neighbour-joining tree based upon concatenated sequences of MLST alleles from human *S. aureus *strains**. MLST allele sequences representing each clinical strain studied here were used to generate a neighbour joining tree using MEGA 4. The A domain isotypes carried by strains of each MLST genotype, determined by sequencing and hybridization analysis, are indicated. The dashed line indicates the separation of the MLST genotypes into Groups 1 and 2, which is based on sequence data from MLST alleles and other unlinked loci [24]. The percentage of replicate trees in which the associated taxa clustered together in the bootstrap test (500 replicates) are shown next to the branches [36,37]. The phylogenetic tree was linearized assuming equal evolutionary rates in all lineages [37]. The evolutionary distances were computed using the Maximum Composite Likelihood method [34]. and are in the units of the number of base substitutions per site.

Figure 3 shows a neighbour-joining phylogenetic tree which was constructed based upon the concatenated sequences of the seven housekeeping genes used for MLST analysis. As MLST reflects the evolution of the stable core genome [23], this tree describes the phylogenetic relatedness of the *S. aureus *strains studied here. It is separated into two major clusters as was also shown previously in a detailed phylogenetic analysis of thirty diverse *S.aureus *isolates [24]. The FnBPB A domain isotypes specified by each genotype (as predicted by DNA hybridisation or sequencing) are indicated. The phylogeny of *fnbB *alleles illustrated here does not correspond to that of the core genome as determined by MLST. For example, two strains that cluster together in Group 1 (ST49 and ST52) carry *fnbB *genes encoding isotype II, as do distantly related strains from Group 2 (ST5 and ST18). Conversely, clustered strains such as ST8 and ST97 from Group 2 contain *fnbB *genes encoding isotypes I and IV, respectively. Isolates belonging to the same ST (ST45) were found to specify different FnBPB isotypes (II and VII). These results suggest that *fnbB *alleles have dispersed by horizontal transfer, most likely by homologous recombination.

It has been recently reported that strains 116 (ST9) and 3077 (ST17) specify an identical FnBPA A domain called isotype II [22]. In this study, these strains were found to specify different FnBPB A domains, isotypes II and VI respectively. This indicates that the phylogeny of *fnbB *alleles does not match that of *fnbA *alleles despite the two genes being closely linked.

### FnBP isotypes encoded by bovine *S. aureus *strains

We expanded the investigation into FnBP variation to include FnBPs from a variety of bovine *S. aureus *strains. Nineteen bovine isolates representing genetically unrelated strains were screened to determine if they specified the same FnBP isotypes as human strains. This strain collection included strain RF122, the genome of which has been sequenced [25]. RF122 contains only one *fnb *gene encoding FnBPA.

DNA encoding *fnbA *was amplified from the genomic DNA of each strain using generic A domain primers. PCR products hybridised to FnBPA probes specific for isotypes I, II, III or IV. Similarly *fnbB *DNA was amplified by PCR from the genomic DNA of all strains except RF122. These PCR products hybridised to FnBPB probes specific for isotype I, II, III, IV or V. These results indicate that the FnBP isotypes which are expressed by human strains are also specified by bovine strains. Furthermore, the results of this study suggest that the lack of *fnbB *in the genome of strain RF122 is not characteristic of all bovine strains. None of the strains tested specify FnBPA or FnBPB isotypes V, VI or VII.

**Figure 4:**
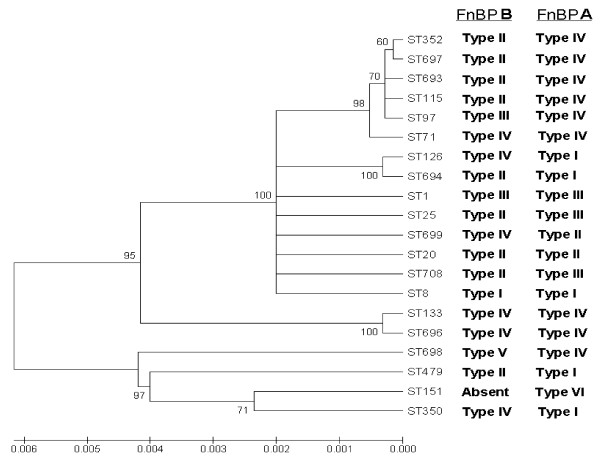
**Neighbour-joining tree based upon concatenated sequences of MLST alleles from bovine *S. aureus *strains**. MLST allele sequences representing each bovine-specific strain studied here were used to generate a neighbour joining tree using MEGA 4. The A domain isotypes carried by strains of each MLST genotype, as determined by hybridization analysis, are indicated. A gene encoding FnBPB is absent from the genome of strain RF122 (ST155). The percentage of replicate trees in which the associated taxa clustered together in the bootstrap test (500 replicates) are shown next to the branches [36]. The evolutionary distances were computed using the Maximum Composite Likelihood method [34] and are in the units of the number of base substitutions per site.

Figure 4 shows a neighbour-joining phylogenetic tree which was constructed based on MLST data as described above. The FnBPA and FnBPB A domain isotypes specified by each genotype are included. The distribution of *fnbB *and *fnbA *variants does not correlate with the genetic relatedness of the strains as determined by MLST. The phylogeny of *fnb *alleles carried by bovine *S. aureus *isolates is therefore very similar to that of human strains.

### Generation of 3D-models for FnBPB (N23) types I-VII and mapping the location of variant amino acid residues

**Figure 5:**
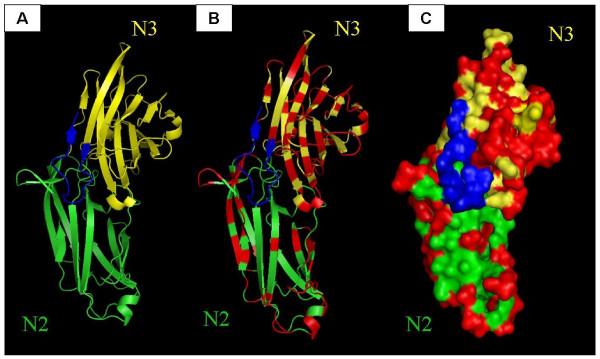
**Predicted 3D Structure of FnBPB isotype I**. Based on the crystal structure of domain A of ClfA, a ligand-binding trench is predicted to form between the N2 (green) and N3 (yellow) domains of FnBPB. The fourteen C-terminal residues that are predicted to form the putative latching peptide are shown in blue. Residues that differ in FnBPB types II, III and IV are highlighted in red in the ribbon (**B**) and space fill (**C**) models. Residues that are predicted to form the latching peptide and ligand binding trench are conserved while variant residues are located on the surface.

Theoretical models of the structure of region A (N23) of FnBPB isotypes I-VII were generated based on the crystal structure of the equivalent domains of the *S. aureus *clumping factor ClfA. A ligand-binding trench is predicted to form between the N2 and N3 domains of FnBPB. C-terminal residues in sub-domain N3 are predicted to form the putative latching peptide. In each of the seven molecular models, the variant residues mapped to the surface of the protein while the residues within the predicted ligand-binding trench are highly conserved (Figure 5.). The predicted 3D structure obtained for FnBPB type I of strain 8325-4 and the predicted location of variant residues is shown in Figure 4. Residues 467-480 of FnBPB isotype I comprise the predicted latching peptide and are shown here in blue. In the crystal structure of the apo form of ClfA the latching peptide is folded over the N3 subdomain.

### Antigenic variation: binding of antibodies to isotypes I-VII

**Figure 6:**
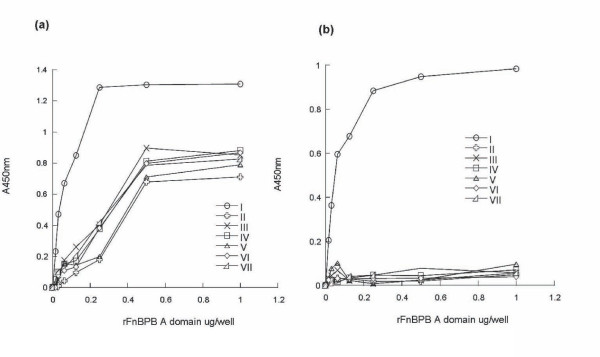
**Binding of polyclonal and monoclonal anti-isotype I A domain antibodies to recombinant A domain isotypes I - VII**. Microtitre dishes were coated with A domains isoype I - VII at the indicated concentrations. Wells were blocked and then incubated with (a) polyclonal rabbit anti-isotype I A domain antibodies, or (b) mouse monoclonal anti-isotype I A domain antibody 2E11. Bound antibodies were detected with either (a) HRP-conjugated goat anti-rabbit IgG antibodies or (b) HRP-conjugated goat anti-mouse IgG antibodies followed by TMB substrate. Graphs are representative of three separate experiments.

We previously demonstrated that variation in the A domain of FnBPA resulted in proteins that are antigenically distinct. Here the ability of polyclonal anti-isotype I antibodies and a monoclonal anti-isotype I antibody to bind different recombinant FnBPB N23 isotypes was measured by ELISA. Polyclonal rabbit anti-isotype I antibodies had a 4 - 9 fold lower affinity at half maximum binding for isotypes II - VII compared to isotype I (Figure 6). This suggests that amino acid variation creates differences in surface-exposed epitopes on the A domain molecule that affect immuno-crossreactivity. Mouse monoclonal antibody 2E11 bound efficiently to isotype I but showed little binding to isotypes II - VII as shown in Figure 5. This suggests that the 2E11 epitope is only present on isotype I.

### Binding of FnBPB A domains isotypes I - VII to immobilized ligands (ELISA)

**Figure 7:**
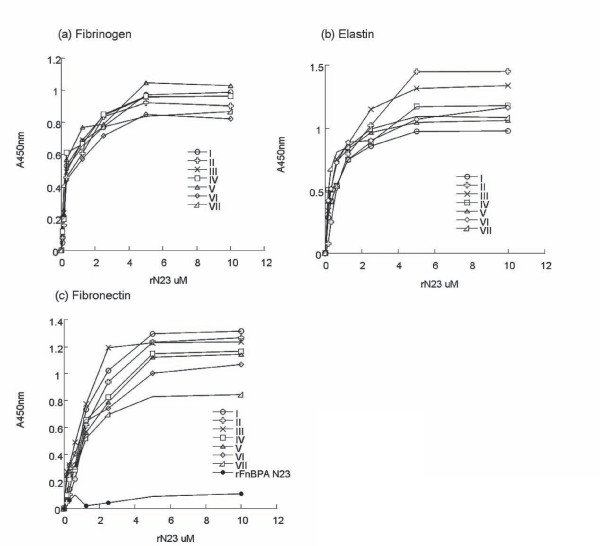
**Dose-dependent binding of rN23 isotypes I-IV to immobilised human fibrinogen (a), elastin (b) and fibronectin (c)**. Bound protein was detected with mouse anti-hexahistidine monoclonal antibody 7E8. rFnBPA N23 was used as a control in fibronectin-binding assays. Each assay was preformed three times with similar results.

Each recombinant N23 isotype bound to immobilized fibrinogen and elastin in a dose-dependent and saturable manner as shown in Figure 7. The estimated half maximum binding concentrations were 0.5 μM and 0.9 μM respectively. These results confirm that the revised co-ordinates of the N23 subdomain of region A of FnBPB (isotypes I-VII) is sufficient for ligand-binding and that subdomain N1 is not required.

Somewhat surprisingly, the seven N23 isotypes also bound fibronectin dose-dependently and saturably with a half-maximum binding concentration of 1.5 μM (Figure 7c). Recombinant FnBPA isotype I, which was previously shown not to bind fibronectin, was a used was as a negative control. The ability of the FnBPB A domain proteins to bind fibronectin was surprising because the amino acid sequences do not contain any known fibronectin-binding motifs.

### Measuring the affinity of FnBPB A domain isotype I for fibrinogen, elastin and fibronectin by surface plasmon resonance

**Figure 8:**
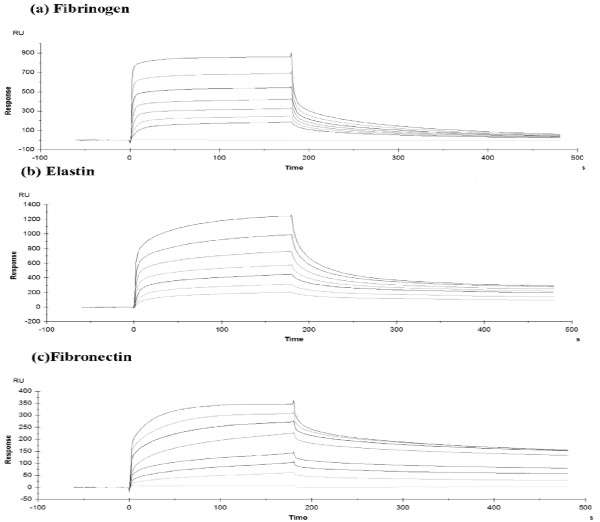
**Dose-dependent binding of rFnBPB to fibrinogen (a), elastin (b) and fibronectin (c) as determined by Surface Plasmon Resonance**. Human fibrinogen, elastin and fibronectin were immobilised onto the surface of dextran chips. In each assay, recombinant FnBPB N23 isotype I was passed over the surface in concentrations ranging from 0.15 μM (lower-most trace) to 10 μM (upper-most trace). The phases of association and dissociation are indicated. The representative sensorgrams have been corrected for the response obtained when recombinant FnBPB proteins were flowed over uncoated chips.

The results of the solid-phase binding assays suggested that the A domain of FnBPB binds fibrinogen, elastin and fibronectin with similar affinity. Estimated half maximal binding concentrations were in the low micromolar range. To verify these results, the affinities of rN23 isotype I for fibrinogen, elastin and fibronectin were measured using Surface Plasmon Resonance. Human fibrinogen, elastin and fibronectin were immobilized onto the surface of dextran chips. rN23 type I protein was passed over the surface in concentrations ranging from 0.15 μM to 10 μM. The representative sensorgrams shown in Figure 8 have been corrected for the response obtained when recombinant protein was flowed over uncoated chips. The K_*D *_for the interaction with fibrinogen, elastin and fibronectin was 2 μM, 3.2 μM and 2.5 μM, respectively.

## Discussion

The colonization of host tissue by *S. aureus *is an important factor in disease pathogenesis. *S. aureus *expresses on its cell surface a number of MSCRAMMS that promote colonization of diverse sites and contribute to virulence. Most *S. aureus *strains can express two distinct fibronectin-binding proteins (FnBPA and FnBPB). These two multifunctional MSCRAMMs both mediate adhesion to fibrinogen, elastin and fibronectin.

FnBPA and FnBPB are encoded by the two closely linked genes, *fnbA *and *fnbB *[20]. It has been reported that the *fnbA *and *fnbB *genes from 50 different strains representing the major MRSA clones found in Europe have undergone greater sequence divergence than genes encoding other surface proteins such as *clfA *and *clfB *[26]. Analysis of the *fnb *genes from published genome sequences showed that divergence was confined to the region encoding the N-terminal fibrinogen and elastin-binding A domains while the C-terminal fibronectin-binding motifs were highly conserved ([22] and this study). Our previous study identified seven isotypes of FnBPA based on divergence in the minimal ligand-binding N23 sub-domain [22]. Each recombinant isotype was found to retain ligand-binding function but was antigenically distinct.

This study aimed to investigate the divergence in the A domain of FnBPB and to determine if variation in this region of the protein is widespread amongst *S. aureus *strains. The *fnbB *gene sequences from sequenced *S. aureus *strains and strain P1 were compared. Four FnBPB variants (isotypes I-IV) were identified based on divergence in N23 sub-domains, which are 66-76% identical to one another.

In order to determine the distribution of FnBPB isotypes I-IV and to identify novel isotypes, type specific probes were generated and used to screen *fnbB *DNA from a variety of clonal types using a well-characterized strain collection of human origin and human isolates where genomes have been fully sequenced [27]. Three novel FnBPB isotypes were identified (types V, VI and VII) which are 61.1% - 85% identical to isotypes I-IV. Phylogenetic analysis of FnBPB isotypes indicated that the phylogeny of *fnbB *alleles does not correlate with the core genome as reflected by MLST. The evolution of *S. aureus *has been predominantly clonal where alleles are 5- to 10-fold more likely to diversify by point mutations than by recombination [27]. The distribution of *fnbB *alleles amongst different *S. aureus *lineages suggests, however, that recombination has been involved. Horizontal transfer by homologous recombination is likely to be responsible for the dispersal of genes encoding the same isotypes across strains of different phylogenies. The distribution of *fnbA *alleles described in the study by Loughman *et al *does not match the distribution of *fnbB *alleles described here [22]. Different combinations of FnBPA and FnBPB isotypes are specified by strains that cluster phylogenetically. For example, strains belonging to ST12 were shown to specify FnBPB Type V and FnBPA Type V. By contrast, strains belonging to ST13 specified FnBPB Type IV and FnBPA Type V. This suggests that the phylogeny of *fnbB *alleles has evolved independently from that of *fnbA *alleles and has involved separate recombination events despite the genes being closely linked.

Our study of FnBP variation in *S. aureus *was extended here to include bovine *S. aureus *strains. The genome of the bovine strain RF122 contains only the *fnbA *gene but lacks *fnbB*. Using generic primers, DNA encoding FnBPA and FnBPB was amplified from genomic DNA of nineteen bovine *S. aureus *strains. The amplification of *fnbB *DNA from these strains indicates that the lack of the *fnbB *gene in strain RF122 is not common to all bovine *S. aureus *strains. The *fnbA *and *fnbB *PCR products were subsequently probed with DNA probes specific for A domain isotypes specified by human *S. aureus *strains. It was shown that bovine isolates specify the some of the same isotypes of FnBPA and FnBPB as those specified by human isolates. The distribution of isotypes across the population of bovine strains tested was found to be uneven. No strains tested specified FnBPA isotypes V, VI or VII or FnBPB isotypes VI or VII. The majority of the strains tested were found to specify FnBPA Type IV and FnBPB Type II. Interestingly in the study of Loughman *et al*, FnBPA Type II was found to be predominant in human clinical isolates [22]. It could be postulated that this difference in FnBPA isotype frequency reflects the differences in selective pressures posed by these two distinct host immune systems.

Further evidence for the role of recombination in the evolution of *S.aureus *comes from the genome structure of ST239 strains which are composed of 557 kb of ST8 DNA spliced into 2,220 kb of an ST30 strain [28]. Also, the gene for coagulase has undergone similar diversification as the *fnb *genes [29]. Recombination within *coa *genes encodeding ten major isotypes has created novel subtypes and there is evidence for the same *coa *isotype being expressed by strains with different genetic backgrounds suggesting horizontal dissemination by homologous recombination [29].

A 3D molecular model of the N2 and N3 domains of FnBPB was generated based on the known structure of ClfA. Like the A domain of ClfA (and FnBPA) it is predicted that the N23 subdomain of FnBPB represents the minimal ligand binding region and a ligand binding trench is predicted to form between the N2 and N3 subdomains. Based on this model, it was shown that the majority of variant residues are located on the surface of the protein while residues that are predicted to be involved in ligand-binding are highly conserved. Amino acid sequence variation affected antibody recognition. Polyclonal antibodies against isotype I had reduced affinity for isotypes II - VII while a monoclonal antibody raised against isotype I had little or no affinity for all other isotypes. As with FnBPA isotypes, FnBPB sequence variation has created different epitopes on the A domains that affect immunocross-reactivity. This result is consistent with the predicted location of variant residues on the surface of the protein and not in regions involved in ligand binding.

While most strains contain both genes, some strains contain only *fnbA *[20]. Studies with site-specific *fnbA *and *fnbB *insertion mutants of strain 8325-4 have shown that either FnBPA or FnBPB can mediate adherence to immobilized fibronectin, but there was no difference in adherence between wild type strains and single *fnb *mutants, indicating functional redundancy [21]. However, isolates associated with invasive diseases are significantly more likely to have two *fnb *genes [20]. Combined antigenic variation in both FnBPA and FnBPB may be employed by *S. aureus *to thwart the host immune responses during colonization or invasive infection. Interestingly, the diversity which occurs in the N2 and N3 subdomains of FnBPA and FnBPB does not occur in the N1 subdomain of either protein. For both FnBP proteins, the N1 subdomain is not required for ligand binding, similar to ClfA [13]. The A domain of both ClfA and another *S. aureus *fibrinogen binding protein, clumping factor B (ClfB), are susceptible to cleavage by aureolysin at a SLAVA/SLAAVA motif located between subdomains N1 and N2 [30]. A SLAVA-like motif occurs in both FnBP proteins with S_177_ADVA_181 _and S_144_TDVTA_149 _present in FnBPA isotype I and FnBPB isotype I, respectively, which may render the A domains similarly susceptible to proteolysis. Perhaps the highly conserved N1 subdomains are less readily recognized by the host immune system and may function to protect the ligand-binding N2N3 during early stages of infection.

The ligand binding ability of recombinant FnBPB N23 subdomain isotypes I-VII was compared by ELISA-based solid phase binding assays. Each A domain isotype bound to immobilized fibrinogen and elastin with similar affinities. These results confirm that like the A domains of ClfA and FnBPA, the N23 subdomain of FnBPB is sufficient for ligand-binding and that the N1 subdomain is not required for ligand-binding. The results also suggest that these ligand-binding functions are biologically important and are consistent with the predicted location of variant residues on the surface of the protein and not in regions predicted to be involved in ligand binding. Using the recombinant N23 isotype I protein as a prototype, the affinity of FnBPB for fibrinogen and elastin was analysed by SPR. The K_*D *_for both interactions was in the low micro molar range.

Somewhat surprisingly, the seven recombinant N23 FnBPB isotypes examined in this study bound immobilized fibronectin with similar affinity. The interaction between rN23 Type I (residues 162-480) was verified by SPR analysis with a K_*D *_in the low micro molar range. The results of these fibronectin-binding assays are surprising because this region of FnBPB does not contain any known fibronectin-binding motifs suggesting that the A domains of FnBPB (isotypes I-VII) contain a novel fibronectin-binding motif and may bind fibronectin by a novel mechanism. Experiments are currently underway to examine the biological significance of fibronectin-binding by the A domain of FnBPB and to determine a mechanism for this interaction and identify the FnBPB binding region(s) in human fibronectin.

## Conclusions

We have identified seven isotypes of the N terminal A domain of FnBPB in a genetically diverse collection of human *S. sureus *strains. Amino acid variation creates differences in immuno-crossreactivity while ligand-binding functions are maintained. This may contribute to immune evasion by *S. aureus*. The distribution of FnBPB isotypes throughout the *S. aureus *population is random but does not correlate with the random distribution of FnBPA isotypes described previously. This suggests that *fnbA *and *fnbB *alleles have been dispersed independently by horizontal transfer which most likely involved homologous recombination. Four of the seven FnBPB isotypes were also identified in bovine *S. aureus *strains. The lack of *fnbB *in strain RF122 is not common to all bovine strains. All seven recombinant A domain isotypes bound fibronectin with a K_*D *_in the low micro molar range. This raises the possibility that the A domain of FnBPB binds fibronectin by a novel mechanism. These data have implications for the FnBPB A domain as a target for a vaccine or immunotherapeutics.

## Methods

### Bacterial strains and growth conditions

*Escherichia coli *strains were cultivated on L-agar and L-broth with shaking at 37°C. Cloning was routinely performed in *E. coli *strain XL-1 Blue (Stratagene). *E. coli *strain TOPP 3 (Qiagen) was used for the expression of recombinant FnBPB A domain proteins. Ampicillin (100 μg ml^-1^) was incorporated into growth media where appropriate. The *Staphylococcus aureus *strains used in this study are listed in Table 2 and were cultivated on trypticase soy agar (TSA) or broth (TSB). Human *S. aureus *strains from individuals from Oxfordshire, U.K have been characterized by multi-locus sequence typing (MLST) [27]. Strain P1 is a rabbit virulent strain [31] and has been characterised by MLST [22]. Bovine *S.aureus *strains were a kind gift from Cyril Smyth (Trinity College, Dublin). They were isolated from geographically diverse locations and were characterized by MLST [32].

**Table 2:** *S. aureus *strains screened for FnBPB isotypes.

Strain	ST	Host	FnBPB	Method of Detection
				
8325-4	8	Human	I	Genome sequence [9]
N315	5	Human	II	Genome sequence [38]
MSSA476	1	Human	III	Genome sequence [39]
P1	973	Rabbit	IV	*fnbB *gene sequence (Genbank: HM196815)
2	7	Human	V	*fnbB *gene sequence (Genbank: HM196814)
19	10	Human	IV	DNA hybridization
114	39	Human	II*	*fnbB *gene sequence (Genbank: HM196816)
116	9	Human	II	DNA hybridization
138	30	Human	II*	*fnbB *gene sequence (Genbank: HM196817)
162	1	Human	III	DNA hybridization
304	39	Human	II*	*fnbB *gene sequence (Genbank: HM196818)
316	49	Human	II	DNA hybridization
402	13	Human	IV	DNA hybridization
563	37	Human	II*	*fnbB *gene sequence (Genbank: HM196819)
617	45	Human	II	DNA hybridization
863	20	Human	II	DNA hybridization
964	18	Human	II	DNA hybridization
3015	123	Human	IV	DNA hybridization
3077	17	Human	VI	*fnbB *gene sequence (Genbank: HM196821)
3084	52	Human	II	DNA hybridization
3089	97	Human	IV	DNA hybridization
3110	12	Human	V	*fnbB *gene sequence (Genbank: HM196820)
3132	2	Human	II	DNA hybridization
233	45	Human	VII	*fnbB *gene sequence (Genbank: HM196822)
PSA5	698	Bovine	V	DNA hybridization
RF79	71	Bovine	IV	DNA hybridization
MSA1007	708	Bovine	II	DNA hybridization
DS37	20	Bovine	II	DNA hybridization
DS40	1	Bovine	III	DNA hybridization
DS42	479	Bovine	II	DNA hybridization
MSA915	115	Bovine	II	DNA hybridization
MSA1547	699	Bovine	IV	DNA hybridization
MSA1047	350	Bovine	IV	DNA hybridization
DS70	697	Bovine	II	DNA hybridization
MSA1363	25	Bovine	II	DNA hybridization
RF26	97	Bovine	III	DNA hybridization
DS35	696	Bovine	IV	DNA hybridization
MSA1006	8	Bovine	I	DNA hybridization
MSA17.1	693	Bovine	II	DNA hybridization
MSA1011	352	Bovine	II	DNA hybridization
RF283	133	Bovine	IV	DNA hybridization
MSA1468	694	Bovine	II	DNA hybridization
DS36	126	Bovine	IV	DNA hybridization
RF122	151	Bovine	absent	Genome sequence [25]

### Genetic techniques

Plasmid DNA was isolated using the Wizard^® ^Plus SV Miniprep kit (Promega) according to manufacturer's instructions and finally transformed into *E. coli *XL1-Blue cells using standard procedures [33]. Chromosomal DNA was extracted using the Bacterial Genomic DNA purification kit (Edge Biosystems). Restriction digests and ligations were carried out using enzymes from New England Biolabs and Roche according to the manufacturers' protocols. Oligonucleotides were purchased from Sigma-Genosys and are listed in Table 3. DNA purification was carried out using the Wizard^® ^SV Gel and PCR Clean-up System (Promega).

**Table 3:** Primers.

Flanking primers	
pfnbB Adom F	CCG*GGATCC*AAGAAAACACAAATTGGGAGC
pfnbB Adom R	CCG*GGATCC*ACATGAATAGAATCTTCTTCAG
pfnbA Adom F	CCG*AAGCTT*GTGAAAAACAATCTTAGGTAC
pfnbA Adom R	CCG*GGATCC*TATCAATAGCTGATGAATCCG
	
**Type-specific probe primers**	
pfnbB N3 I F	CTGGTCAAGTAACTAAAGG
pfnbB N3 I R	GTATAATAATAGTTATAATATC
pfnbB N3 II F	ACTGGTCAAGTAACATCTG
pfnbB N3 II R	GTAGTATTTATGATATCCTGA
pfnbB N3 III F	TAAAGGTGGATTGTATACAG
pfnbB N3 III R	TAATAGTAATAACCGTAATTAG
pfnbB N3 IV F	ACTGGTCAAGTAACATCTG
pfnbB N3 IVR	AGTAATAGTTATAATAACCTTG
pfnbB N3 V F	CTGGTCAAGTAACATCTGG
pfnbB N3 V R	GGATAATATGGGTAATAATAGT
pfnbB N3 VI F	GCTAATAAGCCAACAGTCAAAG
pfnbB N3 VI R	CTCGTATATCCAGTTCAATTAACTTG
pfnbB N3 VII F	ATATAAACACATTGGTTCAGATG
pfnbB N3 VII R	TCTCCACTGGAGGCTCAGATTTAATGTC
	
**pQE30 vector primers**	
pfnbBpQE I F	GGG*GGATCC*GGTACAGATGTAACAAATAAAG
pfnbBpQE I R	AAT*CCCGGG*TTACTTTAGTTTATCTTTGCCG
pfnbBpQE II F	GGG*GGATCC*GGTACAGATGTAACAAATAAAG
pfnbBpQE II R	GCG*CCCGGG*TTATTTGGTTTATCTTTACCATCG
pfnbBpQE III F	CCT*GGATCC*GGTACAGATGTAACAAGTAAAGTG
pfnbBpQE III R	AAT*CCCGGG*TTAATTTGGCTTATCTTTACCGTCG
pfnbBpQE IV F	CCT*GGATCC*GGTACAGATGTAACAAATAAAGTG
pfnbBpQE IV R	ATT*CCCGGG*TTAATTTGGCTTATCTTTGCCGTC
pfnbBpQE V F	TAA*GGATCC*GGTACAGATGTAACAAGTAAAG
pfnbBpQE V R	ATT*CCCGGG*TTAATTTGGTTTATCTTTACCGTCG
pfnbBpQE VI F	AAT*GGATCC*GGCTCAGATGTAACAAGTAAAG
pfnbBpQE VI R	TCT*CCCGGG*TTAATTGGGCTTATCTTTGCCGT
pfnbBpQE VII F	CTA*GGATCC*GGTACAGATGTAACAAGTAAAG
pfnbBpQE VII R	AAT*CCCGGG*TTTCTTCGATTGTACCATTC

* restriction endonuclease sites are italicised.

### Cloning of *fnbB *gene fragments

Generic primers, corresponding to conserved DNA encoding the signal sequence and fibronectin binding domain 2, were designed from conserved sequences in *fnbB *genes from publicly available *S. aureus *genomes. PCR products were cleaved with *BamHI *restriction sites incorporated into the primers, ligated to *BamHI-*cleaved pBluescript DNA and transformed into *E. coli*. The cloned *fnbB *gene fragments were sequenced using T3 and T7 primers by GATC Biotech AG (Germany).

### DNA hybridisation using *fnbB *type-specific probes

DIG-labelled isotype-specific probes were synthesised by PCR. Primers were designed to amplify a small region of DNA (~300 bp) in the N3 sub-domain of isotypes I-VII. The PCR products were labelled by incorporating DIG-labelled dNTPs (Roche). Five ng of DNA encoding the A domain of FnBPB from clinical isolates was spotted onto positively charged nylon membranes (Roche) and allowed to air-dry. Membranes were incubated for 5 min on blotting paper soaked in denaturation solution (1.5 M NaCl, 0.5 M NaOH), 5 min in neutralization solution (1.5 M NaCl, 1 M Tris-HCl, pH 7.4), and finally for 15 min on blotting paper soaked with 2× SSC solution (300 mM NaCl, 30 mM tri-sodium citrate). DNA was fixed on the membranes by incubation at 120°C for 30 min. Membranes were incubated for 2 h at 68°C in pre-hybridization solution (5× SSC, 0.1% w/v N-lauroylsarcosine, 0.02% w/v SDS and 1× Blocking Reagent (Roche). DIG-labelled probes were denatured by heating at 95°C for 10 min, diluted in pre-hybridization solution and incubated with nylon membranes for 18 h at 68°C. Following hybridization, the membranes were washed twice with 2× SSC/0.1% w/v SDS at room temperature followed by two washes with 0.5× SSC/0.1% w/v SDS at 68°C for 20 min. Membranes were equilibrated for 30 min in maleic acid buffer (100 mM maleic acid, 150 mM NaCl, pH 7.5), and bound DIG-labelled probes were detected by incubation for 30 min with alkaline phosphatase-conjugated anti-DIG antibody (Roche) diluted 1:10,000 in maleic acid buffer. After washing twice with maleic acid buffer containing 0.3% v/v Tween 20, the chemiluminescence substrate CSPD (Roche) was used to detect bound anti-DIG antibodies and membranes were exposed to X-OMAT UV Plus Film (Kodak).

### Bioinformatic and phylogenetic analysis of FnBPB A domain isotypes

Protein sequences were aligned in pairwise combinations to calculate amino acid identity using the ExPASY SIM alignment tool http://www.expasy.org/tools/sim-prot.html. The concatenated MLST allele sequences of *S. aureus *strains were downloaded from the MLST database http://saureus.mlst.net/. A phylogenetic analysis of concatenated MLST allele sequences was conducted using MEGA version 4 [34]. Alignments of multiple protein sequences to view areas of conservation amongst A domains were performed using Clustal W http://www.ebi.ac.uk/

### Generation of 3D-models for FnBPB (N23) types I-VII

Theoretical models of the structure of region A (N23) types I-VII were obtained by submitting the amino acid sequences for this segment of each protein to the Phyre service of the 3D-PSSM website http://www.sbg.bio.ic.ac.uk/phyre/. This web-based tool models the structure of these sequences based structure of the equivalent domains of the *S. aureus *clumping factor ClfA. All structures were viewed using the pyMOL viewing software.

### Expression of recombinant FnBPB A domain proteins

Primers were designed to amplify DNA encoding residues 162-480 (N23 sub-domain) of FnBPB isotype I from strain 8325-4 by PCR. The primers included *BamHI *and *SmaI *restriction sites to facilitate cloning into the multiple cloning site of the N-terminal six-histidine tag expression vector pQE30 (Qiagen) and incorporated a 3' stop codon. The equivalent N23 regions of FnBPB isotypes types II-VII were PCR-amplified from strains N315, MSSA476, P1, 2, 3077 and 233, respectively. The PCR products were cloned separately into pQE30 and transformed into *E. coli *cells for protein production. Each construct was verified by sequencing (GATC Biotech AG, Germany) and proteins were purified by Ni^2+ ^chelate chromatography [35]. Concentrations were determined using the BCA Protein Assay Kit (Pierce). Proteins were dialysed against PBS for 24 h at 4°C, aliquoted and stored at -70°C.

### Direct binding of recombinant FnBPB A domain proteins to immobilized elastin, fibrinogen and fibronectin

Human aortic elastin (Elastin Products Company; 50 μg/ml) was coated onto microtiter wells for 18 hr under UV light. Wells coated with human fibrinogen (Calbiochem; 10 μg/ml), and fibronectin (Calbiochem; 10 μg/ml) were placed at 4°C overnight. All plates were blocked with 5% skimmed milk in phosphate buffered saline (PBS) for 2 hr at 37°C. Following three washes with PBS containing 0.05% v/v Tween 20 (PBST) various concentrations of purified rFnBPB N23 constructs in PBS were added and incubated at 37°C for 2 hr. After three washes with PBST, bound protein was detected by incubation with a 1:500 dilution of monoclonal antibody 7E8 that recognizes the N-terminal hexahistidine fusion tag. After 1 h incubation with shaking at room temperature, the wells were washed three times with PBST followed by 100 μl per well of goat-anti-mouse IgG antibodies conjugated to horseradish-peroxidase (HRP, Dako; Denmark) diluted 1:2000. After incubation for 1 h at room temperature, wells were washed three times with PBST, and bound HRP-conjugated antibodies were detected with 10 μg per well of 3,3',5,5'-tetramethylbenzidine (TMB; Sigma) in 0.05 M phosphate-citrate buffer containing 0.006% (v/v) hydrogen peroxide. After incubation at room temperature for 5 min the reaction was stopped by adding 50 μl of 2 M H_2_SO_4_. The absorbance at 450 nm was measured with an ELISA plate reader (Multiskan EX, Labsystems).

The purity of the commercial fibronectin used in these assays was examined by SDS-PAGE. ELISA experiments with anti-fibrinogen antibodies revealed that the fibronectin was free of fibrinogen contamination.

### ELISA assays

Various concentrations of recombinant FnBPB A domain proteins in PBS were coated onto Nunc 96-well microtitre dishes for 18 h at 4°C. Wells were washed and blocked with BSA for 2 h as described above. Following three washes with PBST, 100 μl of anti-FnBPB A domain antibodies diluted in BSA-PBST (1.8 μg polyclonal IgG ml^-1^; 2.5 μg monoclonal IgG ml^-1^) were added to each well and incubated for 1 h at room temperature with shaking. Polyclonal antibody raised against the isotype I N23 domain of FnBPB was obtained by immunizing specific pathogen-free rabbits with rFnBPB37-480 from *S. aureus *8325-4. Monoclonal antibody 12E11 was generated by immunizing mice with recombinant isotype I FnBPB37-480. After 1 h incubation the wells were washed three times with PBST. Goat anti-rabbit IgG-HRP conjugated antibodies or goat anti-mouse IgG-HRP conjugated antibodies (Dako, Denmark), each diluted 1:2000 in BSA-PBST, were added to the wells and incubated for 1 h. After washing three times with PBST, bound HRP-conjugated antibodies were detected as described above.

### Analysis of fibrinogen, elastin and fibronectin binding by surface plasmon resonance

Surface plasmon resonance (SPR) was preformed using the BIAcore ×100 system (GE Healthcare). Human fibrinogen (Calbiochem), aortic elastin (Enzyme Research Laboratories) and fibronectin (Calbiochem) were covalently immobilized on CM5 sensor chips using amine coupling. This was performed using 1-ethyl-3-(3-dimethylaminopropyl) carbodiimide hydrochloride (EDC), followed by *N*-hydroxysuccinimide (NHS) and ethanolamine hydrochloride, as described by the manufacturer. Fibrinogen (50 μg/ml), elastin (50 μg/ml) and fibronectin (50 μg/ml) were dissolved in 10 mM sodium acetate at pH 4.5 and immobilized on separate chips at a flow rate of 30 μl/min in PBS (Gibco). Each chip contained a second flow cell, which was uncoated to provide negative controls. All sensorgram data presented were subtracted from the corresponding data from the blank cell. The response generated from injection of buffer over the chip was also subtracted from all sensorgrams. Equilibrium dissociation constants (Kd) were calculated using the BIA ×100 evaluation software version 1.0.

## Authors' contributions

FMB carried out cloning of *fnbB *genes for sequencing and protein expression, DNA and amino acid sequence analysis, *fnbB *DNA hybridisation experiments, phylogenetic analysis, purification of recombinant A domain proteins, ELISA experiments, SPR experiments and drafted the manuscript. NMC carried out *fnbA *DNA hybridization experiments involving bovine *S. aureus *strains. PS and SR were responsible for production of polyclonal and monoclonal antibodies against the isotype I A domain. TJF conceived and coordinated the study, and helped to draft the manuscript. All authors read and approved the final manuscript.
